# Bis[2-(2-pyridylmethyl­eneamino)benzene­sulfonato-κ^3^ *N*,*N*′,*O*]zinc(II) dihydrate

**DOI:** 10.1107/S1600536808026342

**Published:** 2008-08-23

**Authors:** Cheng-Xiang Cai, Miao Ou-Yang, Zhi-Yuan Zhao, Yi-Min Jiang

**Affiliations:** a College of Chemistry and Chemical Engineering, Guangxi Normal University, Guilin, Guangxi 541004, People’s Republic of China; b Department of Chemistry and Life Science, Baise University, Baise 533000, People’s Republic of China

## Abstract

In the title complex, [Zn(C_12_H_9_N_2_O_3_S)_2_]·2H_2_O, the Zn^II^ ion lies on a crystallographic inversion center and is coordinated by four N atoms and two O atoms from two tridentate 2-(2-pyridylmethyl­eneamino)benzene­sulfonate ligands in a slightly distorted octa­hedral environment. In the crystal structure, the complex forms a two-dimensional network through inter­molecular O—H⋯O and C—H⋯O hydrogen bonds.

## Related literature

For related literature, see: Casella & Gullotti (1981 ▶, 1986 ▶); Jiang *et al.* (2006 ▶); Li *et al.* (2006 ▶, 2007 ▶); Wang *et al.* (1994 ▶); Zhang *et al.* (2004 ▶, 2007 ▶, 2008 ▶); Correia *et al.* (2003 ▶); Zheng *et al.* (2001 ▶); Zhou *et al.* (2004 ▶).
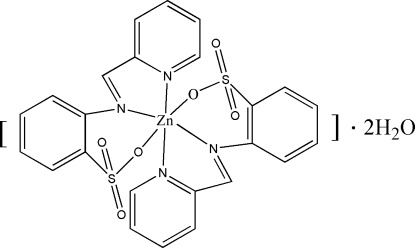


## Experimental

### 

#### Crystal data

[Zn(C_12_H_9_N_2_O_3_S)_2_]·2H_2_O
*M* *_r_* = 623.95
Orthorhombic, 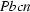

*a* = 19.7090 (15) Å
*b* = 8.0722 (6) Å
*c* = 16.3390 (13) Å
*V* = 2599.5 (3) Å^3^
*Z* = 4
Mo *K*α radiation
μ = 1.16 mm^−1^
*T* = 295 (2) K
0.49 × 0.45 × 0.37 mm

#### Data collection

Bruker SMART CCD area-detector diffractometer
Absorption correction: multi-scan (*SADABS*; Sheldrick, 1996 ▶) *T*_min_ = 0.600, *T*_max_ = 0.673
17894 measured reflections
2412 independent reflections
2100 reflections with *I* > 2σ(*I*)
*R*_int_ = 0.024

#### Refinement

*R*[*F* ^2^ > 2σ(*F* ^2^)] = 0.026
*wR*(*F* ^2^) = 0.074
*S* = 1.04
2412 reflections
177 parameters
H-atom parameters constrained
Δρ_max_ = 0.46 e Å^−3^
Δρ_min_ = −0.42 e Å^−3^

### 

Data collection: *SMART* (Bruker, 2004 ▶); cell refinement: *SAINT* (Bruker, 2004 ▶); data reduction: *SAINT*; program(s) used to solve structure: *SHELXS97* (Sheldrick, 2008 ▶); program(s) used to refine structure: *SHELXL97* (Sheldrick, 2008 ▶); molecular graphics: *SHELXTL* (Sheldrick, 2008 ▶); software used to prepare material for publication: *SHELXTL*.

## Supplementary Material

Crystal structure: contains datablocks global, I. DOI: 10.1107/S1600536808026342/pk2112sup1.cif

Structure factors: contains datablocks I. DOI: 10.1107/S1600536808026342/pk2112Isup2.hkl

Additional supplementary materials:  crystallographic information; 3D view; checkCIF report

## Figures and Tables

**Table 1:** Hydrogen-bond geometry (Å, °)

*D*—H⋯*A*	*D*—H	H⋯*A*	*D*⋯*A*	*D*—H⋯*A*
O4—H1*W*⋯O3^i^	0.83	2.21	3.014 (3)	162
O4—H2*W*⋯O2	0.83	2.06	2.877 (3)	166
C4—H4⋯O3^ii^	0.93	2.48	3.407 (3)	175
C6—H6⋯O4^iii^	0.93	2.57	3.436 (3)	155

Symmetry codes: (i) 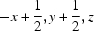; (ii) 
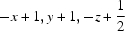
; (iii) 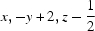.

**Fig. 1.**
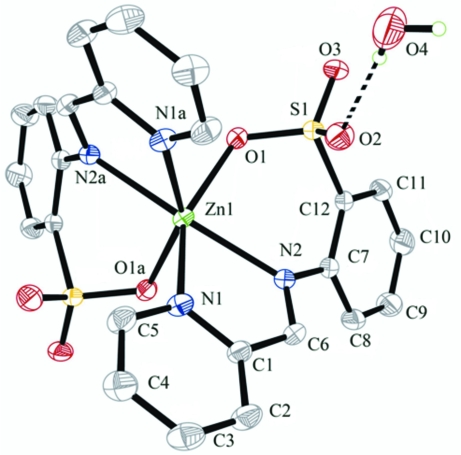
An ellipsoid plot (30% probability) showing the numbering scheme. Dashed lines indicate hydrogen bonds. Symmetry code: (a) -x+1, y, -z+1/2.

**Fig. 2.**
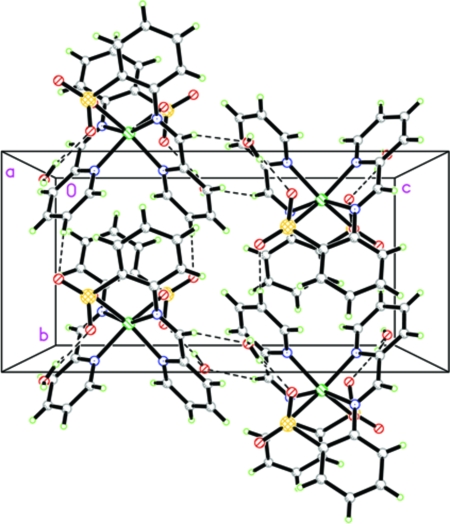
2-D structure, as viewed down the a axis. Dashed lines indicate hydrogen bonds.

## supplementary crystallographic information

### Comment

The design and control of supermolecular coordination complex networks in which both coordination bonds and hydrogen bonds take part in the self-assembly chemistry (Zheng, *et al.*, 2001; Zhou, *et al.*, 2004) have recently garnered increasing interest.  Schiff base complexes that contain both sulfur and amino acid functionalities have received much attention owing to their potential applications in pharmacy. (Casella & Gullotti, 1981, 1986; Wang *et al.*, 1994; Li *et al.*, 2006; Zhang *et al.*, 2007, 2008).

Our group has focused on the exploration of the coordination chemistry of the sulfonate ligands for years (Zhang *et al.* 2004; Jiang *et al.* 2006; Li *et al.* 2007). We report here the synthesis and the structure of the mononuclear Zn^II^ Paba complex (Fig. 1). The structure is composed of one Zn^II^, two deprotonated Paba^-^ ligands and two guest water molecules. The six-coordinated Zn^II^ atom has a distorted octahedral geometry, being coordinated by pyridine N, imine N and sulfonate O atoms from two deprotonated Paba^-^ ligands in a tridentate facial arrangement. This structure is similar to those reported for complexes with N,N',O-tridentate donor ligands (Li *et al.*, 2006; Correia *et al.*, 2003).

There are extensive hydrogen bonds (O4-H2W···O2 and O4-H1W···O3), in which the donor is O-H of the guest water and S=O acts as acceptor, which forms a two-dimension sheet structure (Fig. 2).

### Experimental

The potassium salt of 2-(pyridylmethyl)imine-2-benzenesulfonic acid (PabaK) was synthesized according to the literature method (Casella & Gullotti, 1986).

To prepare the title complex, the ligand PabaK (1 mmol, 0.30 g) was dissolved in methanol (10 mL) at 333 K and an aqueous solution (10 mL) containing ZnCl_2_(0.5 mmol, 0.068 g) was added. The resulting solution was stirred at 333 K for 4 h, then cooled to room temperature and filtered. Yellow crystals suitable for X-ray diffraction were obtained by slow evaporation over several days, with a yield of 55%. Elemental analysis, found (%): C, 46.05; H, 3.55; N, 8.95; S, 10.42; calc (%): C, 46.16; H, 3.53; N, 8.98; S, 10.26.

### Refinement

H atoms bonded to C atoms were positioned geometrically with the C-H distance of 0.93 Å, and treated as riding atoms, with U_iso_(H) = 1.2U_eq_(C). Water hydrogens were placed in fixed positions and assigned U_iso_ values of 1.5U_eq_ of the water oxygen atom.

### Crystal data

**Table:**

[Zn(C_12_H_9_N_2_O_3_S_1_)_2_]·2H_2_O	*F*_000_ = 1280
*M**_r_* = 623.95	*D*_x_ = 1.594 Mg m^−^^3^
Orthorhombic, *P**b**c**n*	Mo *K*α radiation λ = 0.71073 Å
Hall symbol: -P 2n 2ab	Cell parameters from 7323 reflections
*a* = 19.7090 (15) Å	θ = 2.5–28.2º
*b* = 8.0722 (6) Å	µ = 1.16 mm^−^^1^
*c* = 16.3390 (13) Å	*T* = 295 (2) K
*V* = 2599.5 (3) Å^3^	Block, yellow
*Z* = 4	0.49 × 0.45 × 0.37 mm

### Data collection

**Table:**

Bruker SMART CCD area-detector diffractometer	2412 independent reflections
Radiation source: fine-focus sealed tube	2100 reflections with *I* > 2σ(*I*)
Monochromator: graphite	*R*_int_ = 0.024
*T* = 295(2) K	θ_max_ = 25.5º
φ and ω scans	θ_min_ = 2.5º
Absorption correction: multi-scan(SADABS; Sheldrick, 1996)	*h* = −23→23
*T*_min_ = 0.600, *T*_max_ = 0.673	*k* = −9→9
17894 measured reflections	*l* = −19→19

### Refinement

**Table:**

Refinement on *F*^2^	Secondary atom site location: difference Fourier map
Least-squares matrix: full	Hydrogen site location: inferred from neighbouring sites
*R*[*F*^2^ > 2σ(*F*^2^)] = 0.026	H-atom parameters constrained
*wR*(*F*^2^) = 0.074	*w* = 1/[σ^2^(*F*_o_^2^) + (0.0373*P*)^2^ + 1.7627*P*] where *P* = (*F*_o_^2^ + 2*F*_c_^2^)/3
*S* = 1.04	(Δ/σ)_max_ < 0.001
2412 reflections	Δρ_max_ = 0.46 e Å^−^^3^
177 parameters	Δρ_min_ = −0.42 e Å^−^^3^
Primary atom site location: structure-invariant direct methods	Extinction correction: none

### Special details

**Table:**

Geometry. All esds (except the esd in the dihedral angle between two l.s. planes) are estimated using the full covariance matrix. The cell esds are taken into account individually in the estimation of esds in distances, angles and torsion angles; correlations between esds in cell parameters are only used when they are defined by crystal symmetry. An approximate (isotropic) treatment of cell esds is used for estimating esds involving l.s. planes.
Refinement. Refinement of F^2^ against ALL reflections. The weighted R-factor wR and goodness of fit S are based on F^2^, conventional R-factors R are based on F, with F set to zero for negative F^2^. The threshold expression of F^2^ > 2sigma(F^2^) is used only for calculating R-factors(gt) etc. and is not relevant to the choice of reflections for refinement. R-factors based on F^2^ are statistically about twice as large as those based on F, and R- factors based on ALL data will be even larger.

### Fractional atomic coordinates and isotropic or equivalent isotropic displacement parameters (Å^2^)

**Table:**

	*x*	*y*	*z*	*U*_iso_*/*U*_eq_	
Zn1	0.5000	0.82446 (4)	0.2500	0.02769 (11)	
S1	0.37616 (2)	0.67719 (6)	0.34320 (3)	0.03070 (14)	
O1	0.45098 (7)	0.67650 (18)	0.33822 (8)	0.0344 (3)	
O2	0.34865 (8)	0.84229 (19)	0.33446 (10)	0.0456 (4)	
O3	0.35256 (7)	0.5876 (2)	0.41443 (8)	0.0431 (4)	
N1	0.49613 (8)	1.0216 (2)	0.16032 (10)	0.0341 (4)	
N2	0.40820 (8)	0.7687 (2)	0.17245 (9)	0.0297 (4)	
C1	0.44459 (10)	1.0145 (3)	0.10605 (12)	0.0332 (4)	
C2	0.43673 (13)	1.1301 (3)	0.04460 (14)	0.0452 (6)	
H2	0.4007	1.1223	0.0081	0.054*	
C3	0.48322 (13)	1.2579 (3)	0.03809 (15)	0.0506 (6)	
H3	0.4786	1.3380	−0.0025	0.061*	
C4	0.53638 (13)	1.2645 (3)	0.09261 (15)	0.0483 (6)	
H4	0.5688	1.3480	0.0890	0.058*	
C5	0.54084 (12)	1.1447 (3)	0.15295 (14)	0.0420 (5)	
H5	0.5766	1.1503	0.1900	0.050*	
C6	0.39854 (10)	0.8728 (3)	0.11481 (12)	0.0354 (5)	
H6	0.3626	0.8590	0.0786	0.042*	
C7	0.36755 (9)	0.6238 (3)	0.17777 (12)	0.0313 (4)	
C8	0.34696 (11)	0.5353 (3)	0.10925 (13)	0.0437 (5)	
H8	0.3579	0.5736	0.0572	0.052*	
C9	0.31015 (12)	0.3900 (3)	0.11817 (15)	0.0524 (6)	
H9	0.2962	0.3320	0.0720	0.063*	
C10	0.29391 (13)	0.3306 (3)	0.19512 (16)	0.0516 (6)	
H10	0.2698	0.2322	0.2005	0.062*	
C11	0.31364 (11)	0.4180 (3)	0.26416 (13)	0.0403 (5)	
H11	0.3022	0.3792	0.3160	0.048*	
C12	0.35039 (10)	0.5632 (3)	0.25581 (11)	0.0300 (4)	
O4	0.29845 (11)	1.0939 (4)	0.44343 (14)	0.1079 (10)	
H1W	0.2572	1.1144	0.4394	0.162*	
H2W	0.3083	1.0105	0.4158	0.162*	

### Atomic displacement parameters (Å^2^)

**Table:**

	*U*^11^	*U*^22^	*U*^33^	*U*^12^	*U*^13^	*U*^23^
Zn1	0.02732 (18)	0.03263 (19)	0.02311 (18)	0.000	−0.00389 (11)	0.000
S1	0.0291 (3)	0.0396 (3)	0.0234 (2)	−0.0001 (2)	0.00018 (18)	−0.0016 (2)
O1	0.0287 (7)	0.0462 (9)	0.0285 (7)	−0.0028 (6)	−0.0034 (6)	0.0038 (6)
O2	0.0482 (9)	0.0452 (9)	0.0436 (9)	0.0102 (7)	−0.0018 (7)	−0.0079 (7)
O3	0.0416 (8)	0.0620 (10)	0.0257 (7)	−0.0062 (8)	0.0039 (6)	0.0035 (7)
N1	0.0400 (9)	0.0346 (9)	0.0278 (9)	−0.0007 (7)	−0.0057 (7)	0.0011 (7)
N2	0.0278 (8)	0.0375 (9)	0.0238 (8)	−0.0006 (7)	0.0004 (6)	0.0004 (7)
C1	0.0343 (10)	0.0377 (11)	0.0275 (10)	0.0035 (9)	−0.0036 (8)	0.0023 (8)
C2	0.0519 (14)	0.0471 (13)	0.0367 (12)	0.0017 (11)	−0.0113 (10)	0.0096 (10)
C3	0.0728 (17)	0.0386 (13)	0.0403 (13)	−0.0023 (12)	−0.0064 (12)	0.0105 (11)
C4	0.0628 (15)	0.0363 (12)	0.0458 (13)	−0.0117 (11)	−0.0019 (11)	0.0022 (10)
C5	0.0490 (13)	0.0391 (12)	0.0380 (12)	−0.0084 (10)	−0.0102 (10)	0.0012 (9)
C6	0.0310 (11)	0.0475 (12)	0.0276 (10)	0.0001 (9)	−0.0057 (8)	0.0034 (9)
C7	0.0245 (10)	0.0410 (11)	0.0284 (10)	−0.0009 (8)	−0.0004 (8)	−0.0015 (9)
C8	0.0417 (12)	0.0609 (15)	0.0286 (11)	−0.0085 (11)	0.0014 (9)	−0.0050 (10)
C9	0.0505 (14)	0.0679 (16)	0.0389 (13)	−0.0180 (13)	−0.0001 (11)	−0.0169 (12)
C10	0.0464 (14)	0.0552 (15)	0.0530 (15)	−0.0211 (12)	0.0021 (11)	−0.0085 (12)
C11	0.0363 (12)	0.0495 (13)	0.0351 (11)	−0.0088 (10)	0.0038 (9)	0.0017 (10)
C12	0.0232 (9)	0.0399 (11)	0.0270 (10)	−0.0007 (8)	−0.0001 (7)	−0.0023 (8)
O4	0.0592 (13)	0.168 (3)	0.0964 (18)	0.0323 (15)	−0.0209 (12)	−0.0725 (19)

### Geometric parameters (Å, °)

**Table:**

Zn1—O1	2.1065 (14)	C3—C4	1.376 (3)
Zn1—O1^i^	2.1065 (14)	C3—H3	0.9300
Zn1—N1^i^	2.1643 (18)	C4—C5	1.384 (3)
Zn1—N1	2.1643 (18)	C4—H4	0.9300
Zn1—N2	2.2544 (16)	C5—H5	0.9300
Zn1—N2^i^	2.2544 (16)	C6—H6	0.9300
S1—O2	1.4459 (16)	C7—C8	1.389 (3)
S1—O3	1.4470 (15)	C7—C12	1.407 (3)
S1—O1	1.4769 (14)	C8—C9	1.387 (3)
S1—C12	1.7730 (19)	C8—H8	0.9300
N1—C5	1.334 (3)	C9—C10	1.383 (3)
N1—C1	1.350 (2)	C9—H9	0.9300
N2—C6	1.277 (3)	C10—C11	1.386 (3)
N2—C7	1.421 (3)	C10—H10	0.9300
C1—C2	1.380 (3)	C11—C12	1.384 (3)
C1—C6	1.467 (3)	C11—H11	0.9300
C2—C3	1.384 (3)	O4—H1W	0.8332
C2—H2	0.9300	O4—H2W	0.8339
			
O1—Zn1—O1^i^	110.92 (8)	C3—C2—H2	120.5
O1—Zn1—N1^i^	88.27 (6)	C4—C3—C2	118.9 (2)
O1^i^—Zn1—N1^i^	149.76 (6)	C4—C3—H3	120.6
O1—Zn1—N1	149.76 (6)	C2—C3—H3	120.6
O1^i^—Zn1—N1	88.27 (6)	C3—C4—C5	118.9 (2)
N1^i^—Zn1—N1	85.36 (9)	C3—C4—H4	120.6
O1—Zn1—N2	84.45 (5)	C5—C4—H4	120.6
O1^i^—Zn1—N2	82.55 (5)	N1—C5—C4	122.9 (2)
N1^i^—Zn1—N2	123.75 (6)	N1—C5—H5	118.5
N1—Zn1—N2	74.81 (6)	C4—C5—H5	118.5
O1—Zn1—N2^i^	82.55 (5)	N2—C6—C1	119.53 (18)
O1^i^—Zn1—N2^i^	84.45 (5)	N2—C6—H6	120.2
N1^i^—Zn1—N2^i^	74.81 (6)	C1—C6—H6	120.2
N1—Zn1—N2^i^	123.75 (6)	C8—C7—C12	118.80 (19)
N2—Zn1—N2^i^	156.97 (9)	C8—C7—N2	122.62 (18)
O2—S1—O3	114.81 (10)	C12—C7—N2	118.51 (17)
O2—S1—O1	111.87 (9)	C9—C8—C7	120.2 (2)
O3—S1—O1	111.31 (9)	C9—C8—H8	119.9
O2—S1—C12	106.94 (9)	C7—C8—H8	119.9
O3—S1—C12	107.23 (9)	C10—C9—C8	120.7 (2)
O1—S1—C12	103.86 (9)	C10—C9—H9	119.7
S1—O1—Zn1	119.57 (8)	C8—C9—H9	119.7
C5—N1—C1	118.00 (18)	C9—C10—C11	119.9 (2)
C5—N1—Zn1	125.86 (14)	C9—C10—H10	120.1
C1—N1—Zn1	116.11 (14)	C11—C10—H10	120.1
C6—N2—C7	120.22 (17)	C12—C11—C10	119.8 (2)
C6—N2—Zn1	113.76 (14)	C12—C11—H11	120.1
C7—N2—Zn1	125.63 (12)	C10—C11—H11	120.1
N1—C1—C2	122.3 (2)	C11—C12—C7	120.62 (18)
N1—C1—C6	115.76 (17)	C11—C12—S1	120.65 (15)
C2—C1—C6	121.93 (19)	C7—C12—S1	118.73 (15)
C1—C2—C3	119.1 (2)	H1W—O4—H2W	110.2
C1—C2—H2	120.5		

Symmetry codes: (i) −*x*+1, *y*, −*z*+1/2.

### Hydrogen-bond geometry (Å, °)

**Table:**

*D*—H···*A*	*D*—H	H···*A*	*D*···*A*	*D*—H···*A*
O4—H1W···O3^ii^	0.83	2.21	3.014 (3)	162
O4—H2W···O2	0.83	2.06	2.877 (3)	166
C4—H4···O3^iii^	0.93	2.48	3.407 (3)	175
C6—H6···O4^iv^	0.93	2.57	3.436 (3)	155

Symmetry codes: (ii) −*x*+1/2, *y*+1/2, *z*; (iii) −*x*+1, *y*+1, −*z*+1/2; (iv) *x*, −*y*+2, *z*−1/2.
